# Global Coverage Measurement Planning Strategies for Mobile Robots Equipped with a Remote Gas Sensor

**DOI:** 10.3390/s150306845

**Published:** 2015-03-20

**Authors:** Muhammad Asif Arain, Marco Trincavelli, Marcello Cirillo, Erik Schaffernicht, Achim J. Lilienthal

**Affiliations:** Mobile Robotics & Olfaction Lab, Center of Applied Autonomous Sensor Systems (AASS), School of Science & Technology, Örebro University, Örebro SE-70182, Sweden; E-Mails: marco.trincavelli@oru.se (M.T.); marcello.cirillo@oru.se (M.C.); erik.schaffernicht@oru.se (E.S.); achim.lilienthal@oru.se (A.J.L.)

**Keywords:** remote gas detection, mobile robot olfaction, sensor planning, coverage planning, surveillance robots

## Abstract

The problem of gas detection is relevant to many real-world applications, such as leak detection in industrial settings and landfill monitoring. In this paper, we address the problem of gas detection in large areas with a mobile robotic platform equipped with a remote gas sensor. We propose an algorithm that leverages a novel method based on convex relaxation for quickly solving sensor placement problems, and for generating an efficient exploration plan for the robot. To demonstrate the applicability of our method to real-world environments, we performed a large number of experimental trials, both on randomly generated maps and on the map of a real environment. Our approach proves to be highly efficient in terms of computational requirements and to provide nearly-optimal solutions.

## 1. Introduction

In a large number of situations, it is of utmost importance to assess whether a specific gas is present in an area, and establish its concentration, distribution, and the location of the source. This includes the inspection of pipelines and chemical plants to detect the presence of dangerous gas leaks, the longterm monitoring of air pollution levels in big cities or of landfill sites to identify exploitable gas sources. It is often the case that the gases of interest are dangerous for humans and, therefore, human involvement should be minimized.

In the case of pollution monitoring in cities, the deployment of a network of stationary sensors has often proven to be an effective solution [1]. Similar solutions have also been tried in more critical environments, such as coal mines [2] and landfill sites [3]. However, whenever stationary solutions are employed in large areas, two problems arise: the measurements are inherently sparse, so that there can be no guarantee for the absence of the measured gases in the environment (e.g., in a chemical plant a leak could go undetected), and it is very difficult to decide where to place the sensors in the first place, especially if the environment is subject to changes in wind direction and speed, either caused by weather conditions or moving objects.

Mobile robotic olfaction is a recent research direction which aims at overcoming the problems of fixed sensor networks. It combines gas sensors, as used in the fixed networks, with the flexibility of a mobile platform [4]. Different platforms have been successfully tested in recent years for mapping the gas concentration and source localization, both in indoor and outdoor environments [5–7]. The use of mobile platforms has become even more appealing since the introduction of sensors which are capable of detecting gases remotely [8–10]. In particular, sensors based on tunable diode laser absorption spectroscopy (TDLAS) [11,12] allow for ranged sensing up to considerable distances (if the field of view is unobstructed), but are expensive and relatively bulky. Therefore, they are not suitable to be distributed in large numbers in the environment, but they can be used on-board a mobile platform.

The use of ranged sensing devices on mobile platforms introduces new challenges due to the robot's limited autonomy. The largest restriction is posed by battery power and hence the limited duration of monitoring activities. Thereto, it is required to explore the environment *efficiently*. Here, we focus on the problem of *gas detection in large environments*, both outdoor and indoor. We elaborate on the problem of verifying *if* a specific gas is present in the environment or not. More in particular, in this paper we present a novel approach for quickly generating exploration strategies for a mobile robot equipped with a sensor for remote gas detection. The resulting strategies are efficient, in the sense that they minimize the overall time the robot will take to inspect the whole environment.

The contributions of this paper are two-fold. Our first contribution is to formalize the remote gas sensing coverage problem for mobile robotics. Our second contribution is to present a two-step approximate solution to the aforementioned problem which relies on a novel convex optimization-based approximation. Here, we test our solution on large-scale scenarios, to demonstrate its applicability in real-world situations.

The paper is organized as follows: After presenting an overview of relevant work (Section 2), we define and formalize the gas detection coverage problem and how to obtain optimum solutions in Sections 3 and 4. We then present two alternative approaches to decompose the problem, their advantages and drawbacks (Section 5). A first experimental evaluation is detailed in Section 6, where the solution quality and the scalability of the different approaches is discussed. To overcome the scalability issues common to all previous approaches, we present in Section 7 a novel solution which uses re-weighted convex relaxation. We then evaluate this novel solution in Section 8 and conclude in Section 9.

## 2. Related Work

Stationary networks of gas sensors have been used over the years in different environments and in a variety of applications, such as pollution monitoring [13] and the measurement of the concentration of methane in the atmosphere [14]. The sensors commonly employed in such networks are *in situ* [15], which means that they have to come in direct contact with the gas they need to sense. This is obviously a strong limitation: the choice of their positioning is not trivial, new deployments could be necessary whenever the environment changes and the sensors must be re-calibrated and maintained over time. For specific gases, e.g., methane, other viable sensing technologies are available, such as infrared thermography [16], whose results, however, are highly affected by weather conditions, by the nature of the ground surface and by the distance between sensors and sources.

In recent years, many research groups have started to focus on mobile robot olfaction, a solution for gas sensing which would implicitly guarantee more flexibility than sensor networks, as it combines gas sensors with mobile platforms [17,18]. Mobile robots equipped with *in situ* gas sensors have been successfully used for mapping gas distributions [19,20] and leak detection [9,10]. A limitation of current approaches is that the robots generally move between pre-defined positions, or reactively follow gas plumes [21]. Finding good sensing positions for a mobile robot is still an open problem and it is closely related to the sensor placement problem for static networks.

In our work, we do not use *in situ* sensors, but we equip our robot with a TDLAS sensor, which can provide remote measurements up to a well defined sensing range [20]. A sensing action, that is, scanning an area of the environment with our sensor, is expensive, both in terms of time and of battery consumption. Similar costs should be accounted for whenever the robot moves from one sensing position to the next. Since here we are interested in detecting if a specific gas is at all present in a known environment, we need to scan the whole environment. Therefore, if we want to find battery- and time-efficient exploration strategies, we must take into account and minimize the costs associated both to movement and to sensing.

Neglecting the cost associated to the movements of the robot, the problem we would need to solve could be reduced to an *Art Gallery Problem* [22] or to a *View Planning Problem* [23]. The art gallery problem is NP-hard (non-deterministic polynomial-time hard [24]) in its most common variants and view planning is isomorphic to the *Set Covering Problem* [25], a well known NP-complete problem. This family of problems has been extensively studied over the past decades [26,27], but the algorithms proposed that solve the problems optimally are effective under restricted assumptions, such as not considering occlusions in the field of view of the sensors [28], or work only when the number of possible sensing configurations is relatively small [29,30]. There exist, however, efficient algorithms for calculating approximated solutions [31] for problems with a large number of possible sensing configurations. The solutions described above, however, present two major drawbacks in our context: first, they are mostly concerned with camera placement problems, which means that they rarely consider limited field of view; second, they inherently do not consider the cost of moving from one sensor position to the next. On the other hand, assuming known sensing positions, we would still need to solve a (Metric) *Traveling Salesman Problem* (TSP), that is, we should find the shortest tour that connects all the sensing positions. This is a well-known NP-hard problem, but it is possible to optimally solve very large TSP instances, with thousands of locations [32].

Solving an art gallery problem first, and then calculating the shortest tour among the selected sensing positions by solving a TSP is an approach which has proven to be effective in many applications [33–36]. However, all the algorithms proposed rely on simplifying assumptions on the field of view of the sensors (e.g., 360 ° or unrestricted). More important still, the solution of an art gallery problem for a large number of candidate sensing positions with overlapping fields of view remains computationally challenging. By contrast, we use re-weighted convex relaxation to handle a high number of candidate sensing positions.

Our problem can also be related to planning for mobile robotics inspection, although in this case sensing cost is usually neglected. For instance, in [37,38], the authors present an inspection strategy for submerged ship hull with an autonomous underwater vehicle. They address the problem by developing a probabilistic planner to maximize uncertainty reduction while providing coverage of the mesh surface. In our case, we cannot ignore the cost of sensing actions and we aim at guaranteeing that the whole environment is observed. The *Generalized Covering Salesman Problem* [39] can explicitly address this last issue, as a solution of one of its instances is a tour which respects given covering constraints, but still sensing costs are not taken into accounts. A similar problem is solved in [40], where an approach based on mixed integer linear programming is used for finding a surveillance route for a mobile camera. The optimal solution approach we present here is highly reminiscent of the work of Tamioka *et al.* [40] and could be considered an extension of their algorithm: in our case, we can solve larger instances, while also considering costs associated with sensing actions.

A combination of the View Planning Problem and the Metric TSP, the *Traveling View Planning Problem* has been defined and analyzed in [41,42]. It refers to finding a sequence of sensing actions while minimizing the overall cost. Here, the authors use an approximation method to find a bounded suboptimal solution which considers both traveling time and sensing time with respect to the available candidate sensing positions. However, it is not yet clear how to optimally select the candidate sensing positions and the approach presented in these papers does not scale well (in terms of computation time) when the size of the environment grows (in terms candidate sensing positions).

## 3. Problem Definition

In this article, we address the problem of detecting the presence of a specific gas in large environments, both indoor and outdoor. We assume a static environment with no major changes in the average concentration of the target gas due to wind or by other means. This assumption does not imply a major limitation to the gas detection problem, since it is possible to repeat an inspection tour over and over again, in case things are changing. However, these problems need to be addressed for subsequent tasks, like gas source localization and gas distribution mapping as discussed in Section 9.

**Figure 1.**
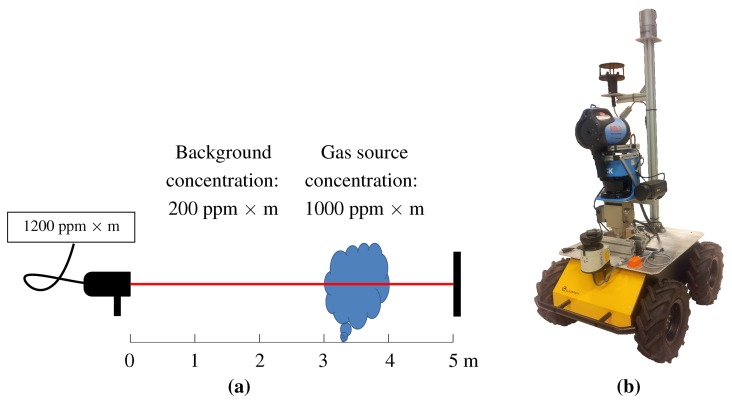
(**a**) the Remote Methane Leak Detector is a TDLAS sensor which can report the integral concentration of methane along its laser beam (parts per million × meter); (**b**) *Gasbot*, a robotic platform for gas detection. Gasbot is a research platform based on a Husky A200. It is specially equipped with a methane sensitive remote sensor (RMLD) mounted in conjunction with a laser scanner on a pan-tilt unit, a laser scanner for self-localization and mapping, an anemometer and a thermal camera.

Furthermore, we assume that the gas, if present, can be detected near ground level. This assumption applies to many real world applications: for instance, methane leaks in a landfill necessarily occur close to the ground. We assume that the presence of the gas in question can be observed by means of a ranged sensor, such as TDLAS. An example of such sensors is the Remote Methane Leak Detector, which can measure the integral concentration of methane over a reflected laser beam (see Figure 1a). The sensor emits light in the near-infrared (NIR) band and analyses the spectrum of the reflection. Each gas has a unique absorption spectrum and the sensor is tuned to the target gas [43]. Hence, the TDLAS technology is very selective and only responds to its target gas even in the presence of multiple airborne substances. Thus, we can safely neglect the complex issue of dealing with gas mixtures. If the utilized sensor is only partially selective, e.g., like the often used metal oxide (MOX) sensors, it would be required to introduce an additional gas discrimination step. Different studies dealing with this particular issue are for example [44–46].

Note that methane is not the only gas that can be detected using this technology. Finally, we assume that the ranged sensor is mounted on a mobile platform, such as the one in Figure 1b, so that its beam can be directed towards the ground at a specific range from the robot and that the sensor can be rotated around its vertical axis.

Given a map of the environment in which we need to assess the presence of a specific gas, we divide it into a Cartesian grid, thus obtaining a set 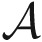 of *n* cells of identical size: 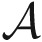 = {*a*_1_, ⋯, *a_n_*}. The set of all cells 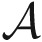 is partitioned into subsets 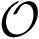 and 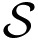, where 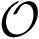 includes all the cells which contain an obstacle and therefore (1) are not traversable by the robot; and (2) can stop the beam of the gas sensor. 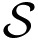 includes all the cells which do not contain obstacles and are therefore traversable by the robot. A solution to the detection problem is an obstacle free closed path, or *tour*, within the map that the robot can traverse and a set of sensing actions along the tour which enables the robot to sense every cell in 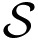.

**Figure 2.**
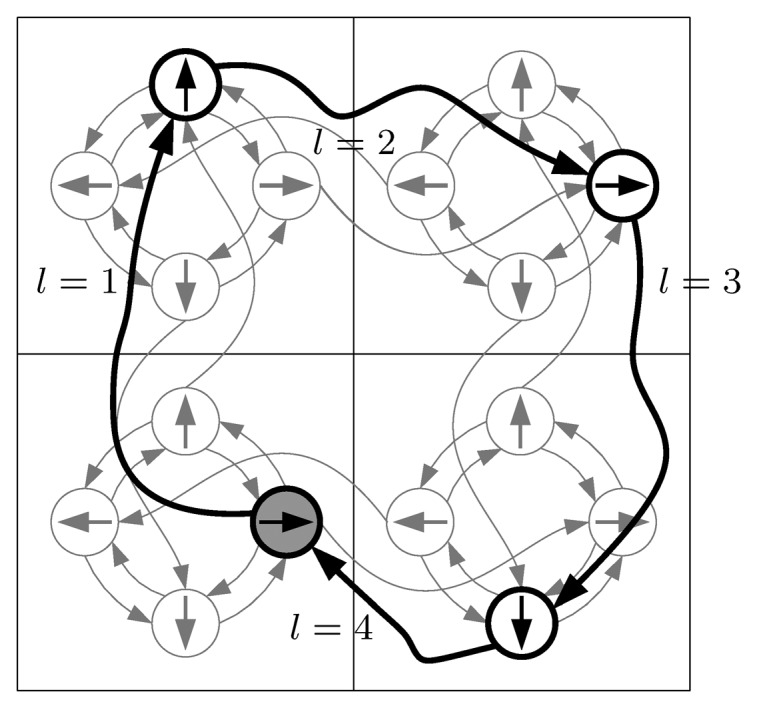
The graph captures the allowed movements of the robot on a grid map when $\Theta =\left\{0,\frac{\pi }{2},\pi ,\frac{3}{2}\pi \right\}$ and only forward movements are allowed. Small circles indicate the poses where the robot can stop (the internal arrow indicates the orientation of the robot) and the directed edges its allowed movements. Note that in the figure the poses do not correspond to the centers of the cells, but this is only for clarity reasons.

We limit the movement of the robot between a finite set of poses 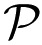. Each *p_j_* ∈ 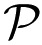 is defined by a two-tuple (*a_j_*, *θ_j_*), where *a_j_* ∈ 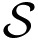 and *θ_j_* ∈ Θ. Θ is a finite set of allowed orientations, equally spaced between [0, 2*π*). Note that the robot position within a cell corresponds to its center, regardless of the orientation. Here, we assume that the movements of the robot are limited to forward motions and rotations. Thus, for $\Theta =\{0,\frac{\pi }{2},\pi ,\frac{3}{2}\pi \}$, the movements of the robot can be captured by a directed graph like the one represented in Figure 2—backward motions and a higher cardinality of Θ could be obviously represented in a similar graph. In our problem definition, we use *time* as measure of cost. Given the times *t^r^*, representing the time necessary to rotate 2*π*/|Θ|, and *t^c^*, the cost associated to moving to the adjacent cell, the movement time $t_{p_{i}\to p_{j}}^{m}$ from *p_i_* to *p_j_* can be easily calculated by finding the shortest path on the movement graph, where each rotation edge has a weight of *t^r^* and every edge leading to a different cell has a weight of *t^c^*. With this decomposition, and with a knowledge of the terrain in each cell, it would be straightforward to express the cost of movement in terms of battery consumption.

We can now define a candidate sensing configuration on the movement graph described above. A candidate sensing configuration corresponds to a possible sensing action of the robot in a specific pose *p*, that is, it represents the possibility of performing a sensing action over a well defined area. Formally:

### Definition 1

*A candidate sensing configuration c_i_ is defined as a tuple* (*p_i_, ϕ_i_, r_i_*)*, where p_i_* ∈ 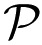 *is the robot's pose, ϕ_i_ is the central angle of a circular sector and r_i_ its radius*.

**Figure 3.**
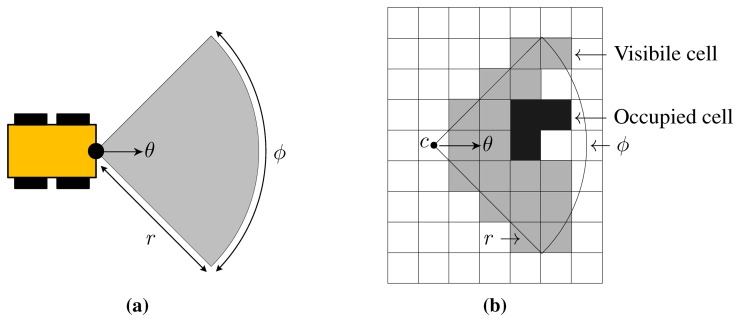
(**a**) a candidate sensing configuration *c* allows the robot to scan a circular sector of central angle *ϕ* and radius *r*; (**b**) *υ*_
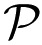
_(*c*) is the visibility function which defines which are the cell that are observable from candidate sensing configuration *c*.

Hence, a candidate sensing configuration *c_i_* would allow the robot positioned in *p_i_* to scan a circular sector of central angle *ϕ_i_* and radius *r_i_*, as shown in Figure 3a. Because of obstructions, not all the cells in the circular sector would be necessarily observable. Also, we need to define which portion of a cell should be swept by the laser beam to consider the cell itself as observed. Let us define 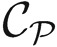 as the set of all candidate configurations defined over a set of robot poses 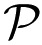. We can then define the visibility function *υ*_
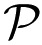
_ : 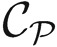 ↦ 2^ℤ^2^^, such that *υ*_
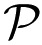
_(*c*) denotes the set of cells ∈ 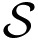 visible from *c*. In the remainder of this paper, we define *υ*_
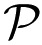
_ so that a cell *k* is considered visible to a sensing configuration *c_i_* if the line segment connecting the centers of *c_i_* and *k* is in the circular sector of *ϕ_i_* and *r_i_*, and does not intersect any occupied cell, as shown in the example in Figure 3b.

The cost associated to perform a sensing action in a candidate sensing configuration *c* is $t_{c}^{s}$ and it depends on the central angle *ϕ* associated with *c*.

## 4. Finding an Optimal Solution

As defined above, given a discretized map of the environment, the sets of occupied and unoccupied cells 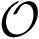 and 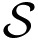 (such that 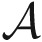 = 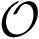 ∪ 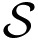), the set of allowed poses 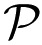 for the robot, the set of candidate sensing configurations 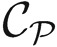 defined over 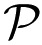, the visibility function *υ*_
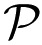
_(*c*) and the cost functions $t_{p_{i}\to p_{j}}^{m}$ (movement time from *p_i_* to *p_j_*, calculated as the shortest path on the movement graph) and $t_{c}^{s}$ (sensing time of candidate configuration *c*), a solution to the detection problem is an obstacle free tour defined as an ordered, finite set of sensing configurations *π* = {*c*_1_, ⋯, *c_k_*} such that ∪*_c_i__*_∈_*_π_ υ*_
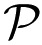
_(*c_i_*) = 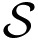. The cost associated to a solution *π* is equal to the sum of the traveling costs (including the traveling cost to go from the last sensing configuration back to the first one) and sensing costs:

(1) $$\text{cost}(\pi )=\sum_{i=1}^{k-1}t_{p_{i}\to p_{i+1}}^{m}+t_{p_{k}\to p_{1}}^{m}+\sum_{c_{i}\in \pi }t_{c_{i}}^{s}$$

Given the set Π of all valid solutions to a given problem instance, an optimal solution *π_opt_* is the one with minimum cost:

$$\pi_{\text{opt}}=\underset{\pi_{i}\in \Pi }{\arg\min}\text{cost}(\pi_{i})$$

### 4.1. Problem Formulation

Practically, we can calculate optimal solutions for the problem defined above by casting it as an optimization problem. Here, we use a formulation inspired by the work of Tomioka *et al.* [40]. More specifically, we define *flow variables* and *label variables* over the movement graph of the robot. Let us consider a problem instance with a set of candidate sensing configurations 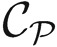. Flow variables are binary variables associated with all pairs of candidate sensing configurations 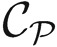. A solution *π* = {*c*_1_, ⋯, *c_k_*} to a problem instance represents a closed walk on the selected candidate sensing configurations. We can then refer to the configurations in a solution *π* by means of their indexes 1, ⋯, *k* and, as we are considering a tour, we can define the configuration of index *k* + 1 = 1, that is, *c_k_*_+1_ = *c*_1_ in *π*. Given a solution *π*, flow variable *f_c_i__*_,_*_c_j__* represents whether *c_i_* and *c_j_* are consecutively traversed in *π*:

(2) $$\begin{array}{cc} \forall c_{i}\in C_{P},\forall c_{j}\in C_{P},c_{i}\neq c_{j} & f_{c_{i},c_{j}}=\{\begin{array}{lll} 1 & \text{if} & c_{i}=c_{h}\in \pi ,c_{j}=c_{h+1}\in \pi .\\ 0 & & \text{otherwise}. \end{array} \end{array}$$

Label variables *l_c_i__*_,_*_c_j__* are non-negative, real variables. They are assigned to the shortest path on the movement graph between any two candidate sensing configurations 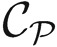. As detailed in [40], label variables enforce flow conservation constraints, that is, constraints which guarantee that a valid solution to a problem instance is composed by closed walks in the movement graph. Given a solution *π* = {*c*_1_, ⋯, *c_k_*}, label variables are assigned in such a way that, given *c_i_* ∈ *π*, the label variable assigned to the (shortest) path between the predecessor in *π* of *c_i_* has a lower value than the one assigned to the (shortest) path between *c_i_* and its successor in *π*. This rule has a single exception in the *special configuration*, or *special vertex*, which is unique for each solution *π*. All other label variables are set to 0. Formally:

(3) $$\forall c_{i}\in C_{P}\;\{\begin{array}{ll} l_{c_{h},c_{i}}<l_{c_{i},c_{j}} & \text{if}\;c_{i}=c_{g}\in \pi ,c_{h}=c_{g-1}\in \pi ,c_{j}=c_{g+1}\in \pi \\ l_{c_{h},c_{i}}>l_{c_{i},c_{j}} & \text{if}\;c_{i}=c_{g}\in \pi ,c_{h}=c_{g-1}\in \pi ,c_{j}=c_{g+1}\in \pi ,c_{i}\;\text{special vertex of}\pi \\ l_{c_{h},c_{i}}=l_{c_{i},c_{j}}=0 & \text{otherwise} \end{array}$$

**Figure 4.**
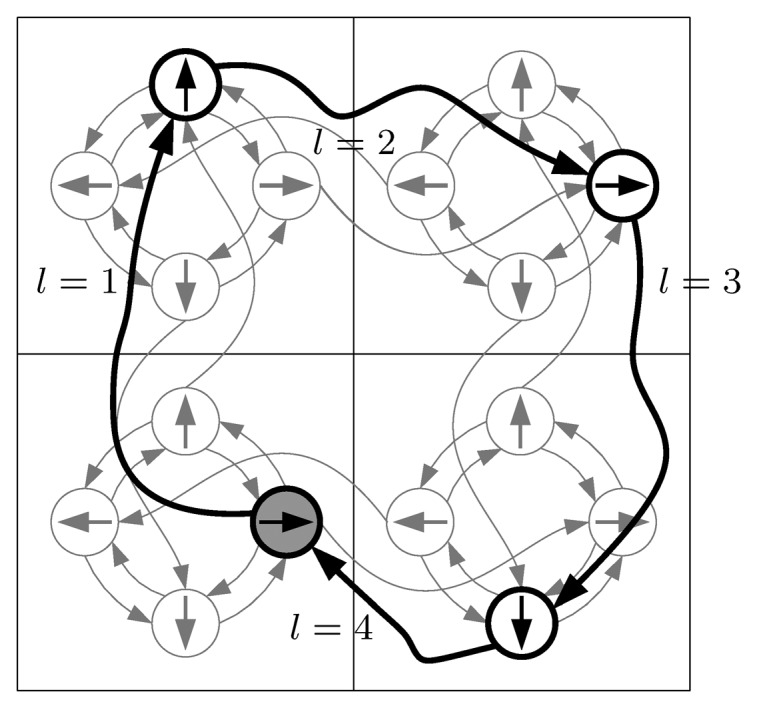
An example of a solution to a problem instance. Configurations in the solution are represented by thick circles and the shortest paths between them by thick lines. Each path between two consecutive candidate sensing configurations in the solution is annotated with a label variable *l*, and the marked configuration corresponds to the special vertex.

Finally, special vertex variables *υ_c_i__* are binary variables defined over every candidate sensing configuration in 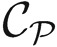, where *υ_c_i__* = 1 if configuration *c_i_* is the unique special vertex in a given solution, *υ_c_i__* = 0 otherwise. An example of a solution *π* where the shortest paths between configurations are annotated with label variables is shown in Figure 4.

We can find an optimal solution to a problem instance by casting it as a mixed integer linear programming problem, which we define as described in Equation (4). Given the set of candidate sensing configurations 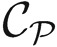, flow and label variables are represented in matrices *F* and *L*, both of size | 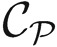
| × | 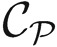
|, where *F*[*i*, *j*] and *L*[*i*, *j*] represent flow variable *f_c_i__*_,_*_c_j__* and label variable *l_c_i__*_,_*_c_j__*, respectively. Please note that |*A*| denotes the cardinality of *A*. Special vertex vector *S* is a boolean vector of size | 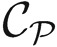
| with each element corresponding to a configuration in 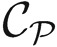.

(4a) $$\begin{array}{l} \mathrm{minimize}1^{T}(F\circ T_{m})1+1^{T}(FT_{s})\\ \text{subject to} \end{array}$$

(4b) (VF)1≽1

(4c) $$F1={\left(1^{T}F\right)}^{T}$$

(4d) F≼L≼uF

(4e) $$-\text{u}S≼\left(\left(L1-{\left(1^{T}L\right)}^{T}\right)-F1\right)≼-S$$

(4f) $$1^{T}S=1$$

The objective function (Equation (4a)) minimizes total cost, expressed as the sum of traveling cost and sensing cost. In this function, matrix *T_m_* of size | 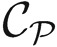
| *×* | 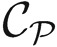
| captures the traveling costs ( $T_{m}[i,j]=t_{p_{i}\to p_{j}}^{m}$) and *T_s_* is a column vector of size | 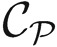
| represents the sensing cost associated to each candidate sensing configuration ( $T_{s}[i]=t_{c_{i}}^{s}$).

The optimization problem is subject to the following constraints:

- **Coverage constraints** (Equation (4b)) require that each cell *a_i_* ∈ 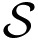 is visible from at least one of the candidate sensing configurations selected in the solution *π*. Given the problem definition, we can calculate *V* as a binary matrix of size *n* × | 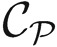
|, where *n* is the number of cells in the problem. In particular:
  $$V[a,c]=\{\begin{array}{cc} 1 & \text{if}\;a\in \upsilon_{P}(c)\\ 0 & \text{otherwise} \end{array}$$
- **Flow conservation constraints** (Equation (4c)) ensure that the solution will consist of one or more closed paths among the selected candidate sensing configurations.
- **Traveling route constraints** (Equations (4d) and (4e)) ensure that each candidate sensing configuration is visited only once in a solution, and, therefore, a solution consists of a single closed path. u is an upper limit constant for label variables.
- **Special vertex constraint** (Equation (4f)) restricts the number of special vertices to one and only one for each solution.

**Figure 5.**
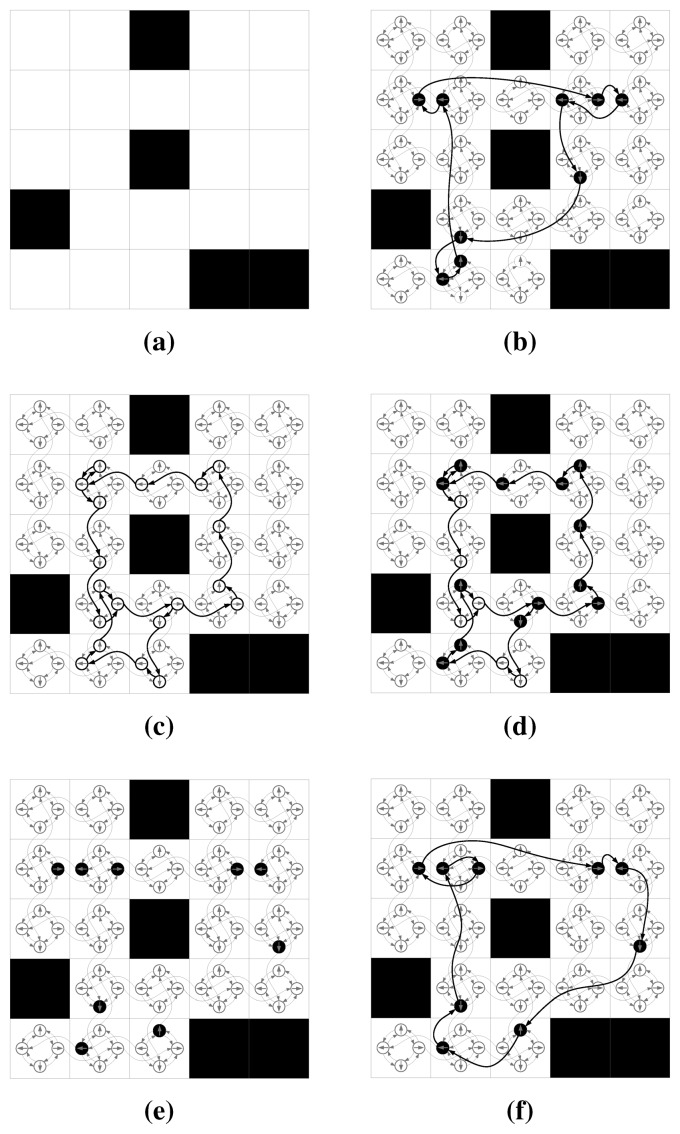
(**a**) a simple test map, where obstructed cells are represented in black and traversable ones in white. Candidate sensing configurations are defined over poses in the cells such that $\Theta =\left\{0,\frac{\pi }{2},\pi ,\frac{3}{2}\pi \right\}$ and have identical *ϕ* and *r* (*r* = 2 cells, $\phi =\frac{\pi }{2}$). In this example setup, the movement from one cell to the next requires 1 s, the rotation of $\frac{\pi }{2}$ requires 0.5 s, and a sensing action takes 4 s; (**b**) the optimal solution, when traveling and sensing costs are considered at the same time. Here, the curved arrows represent the minimum distance from a sensing configuration to the next on the underlying graph. In this case, the total exploration time is 52.5 s (16.5 s for traveling and 36 s for sensing). (**c,d**): Here, traveling time is minimized first (see Section 5.1). (c) the minimum cost closed path from which all cells can be observed is calculated; (d) and then the minimum set of sensing configurations is selected, yielding an overall exploration time of 66.5 s. (**e,f**): Here, sensing time is minimized first (see Section 5.2). (e) the set of minimum cost sensing configurations is selected from which all cells are observable; (f) and then connecting them with the shortest closed path. This approach yields to an overall exploration time of 55 s.

To reduce the size of the problem, without affecting the quality of the solution, it is possible to identify and disregard all those candidate sensing configurations which would not, in any case, belong to an optimal solution. For instance, all those sensing configurations *c_i_* that do not observe any other cells *υ*_
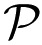
_(*c_i_*) = ∅ (for instance, the ones placed in the cells at the border of the map; can be safely disregarded. Figure 5a is an example test map whose optimal solution is given in Figure 5b.

Solving the mixed integer linear programming problem as described above yields an optimal solution to a given problem instance, with respect to the total operation time of the robot (traveling and sensing time). However, intuitively speaking, this corresponds to solving a combination of a *Watchman Route Problem* (that is, the problem of computing the shortest route to guard a known area) and an *Art Gallery Problem* (that is, selecting the minimum number of observation points to completely observe a known area). Therefore, the combined problem is NP-hard and, as shown in Section 6, this approach becomes practically unfeasible as the number of candidate sensing configurations grows.

## 5. Decomposing the Problem

Solving the problem so that the overall exploration time is minimized is of no practical applicability. However, a viable solution would be to sacrifice optimality by solving the problem in a two-step approach. This can be done in two different ways: (1) minimizing the overall traveling time (while guaranteeing complete coverage) and then selecting the sensing configurations along the route for minimum sensing time; or, (2) minimizing the sensing time, by selecting the sensing configurations necessary to cover the map and then finding the shortest path to connect them. In this section, we formally define such approaches.

### 5.1. Minimizing the Traveling Time

The core idea behind this decoupled approach is to find a minimum cost closed path for the robot which connects adjacent candidate sensing configurations. This path is subject to the requirement that all cells in 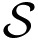 are visible by at least one candidate sensing configuration included in the path. This first step roughly corresponds to solving a Watchman Route Problem. The second step of this approach consists of selecting the minimum cost sensing configurations which would allow the coverage of the whole environment among the ones included in the path.

Intuitively, this approach should yield good results whenever the time required for sensing actions is significantly less than the one for movement. Figure 5c,d show an example of this two phase approach, where first a tour is calculated, such that it connects contiguous candidate sensing configurations from which the whole environment is observable (Figure 5c). Then, in the second phase, the algorithm selects the set of minimum cost candidate sensing configurations on the path necessary to observe the whole environment (Figure 5d). The robot will follow the tour as generated in the first phase, but will only stop for sensing according on those sensing configurations selected in the second phase.

We use the mixed integer linear programming problem as defined by Tomioka *et al.* [40] to find a traveling path that provides full coverage without considering sensing cost. In order to then select the set of minimum cost candidate sensing configurations, we solve a sensor placement problem where the only variables considered are the candidate sensing configurations included in the traveling route. The formulation we use to solve the sensor placement problem is presented in the next section.

### 5.2. Minimizing the Sensing Time

The core idea behind this approach is dual with respect to the one presented above and consists in first selecting a set of minimum cost candidate sensing configurations from which all cells in 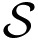 are visible, and then solving a traveling salesman problem over them to find the shortest tour. This approach should therefore yield to better solutions than the previous one whenever the time necessary to perform sensing actions is much higher compared to the time required for the robot to move.

Figure 5e,f show how this approach would work compared to the ones described above: First the set of minimum cost candidate sensing configurations is selected (Figure 5e), and then a shortest closed path is calculated so that the robot will visit all the configurations (Figure 5f).

We find the set of minimum cost candidate sensing configurations by solving the following integer linear programming problem:

(5a) $$\begin{array}{l} \underset{C}{\mathrm{minimize}}C^{T}T_{s}\\ \text{subject to} \end{array}$$

(5b) VC≽1

(5c) C∈{0,1}

where *C* is a column vector of cardinality | 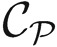
| whose elements are binary variables representing if a given candidate sensing configuration is selected or not. Equation (5b) captures the necessary coverage constraints. For calculating the minimum traveling route, we use the Repetitive Nearest-Neighbor Algorithm [47] for calculating a Hamiltonian cycle.

## 6. Experimental Evaluation

We present an evaluation of the three approaches described above: the one in which traveling time and sensing time are minimized at the same time (Section 4), and the decoupled ones, in which either traveling time or sensing time are minimized first (Section 5). The evaluation is conducted in sets of randomly generated maps of varying size, whose cells have a fixed side length of 1 m. Candidate sensing configurations are defined over poses in the cells such that $\Theta =\{0,\frac{\pi }{2},\pi ,\frac{3}{2}\pi \}$ and have identical *ϕ* and *r* (*r* = 15 meters, $\frac{\pi }{2}$). Sensing and movement times are set based on our previous experience with our mobile platform [20]: Each sensing action takes 4 s, movement from one cell to the adjacent towards which the robot is oriented requires 1 s, while a rotation of the mobile platform of $\frac{\pi }{2}$ requires 0.5 s.

For the first part of the experimental evaluation, we prepared 6 sets of 10 randomly generated maps and one truncated set. Each set contains maps of identical size, starting from small 4 × 4 cell maps, up to 9 × 9 cell maps of which 10% cells contain an obstacle. For 3 × 3 cells there are only three unique maps with a single occupied cell (approx. 10%), all other maps are just rotations. Thereto, the results for 3 × 3 maps are averaged using the unique 3 maps only. Furthermore the maps are checked, whether the observable area is a single connected component. This means, there are no enclosed spaces, which cannot be reached by the robot.

**Figure 6.**
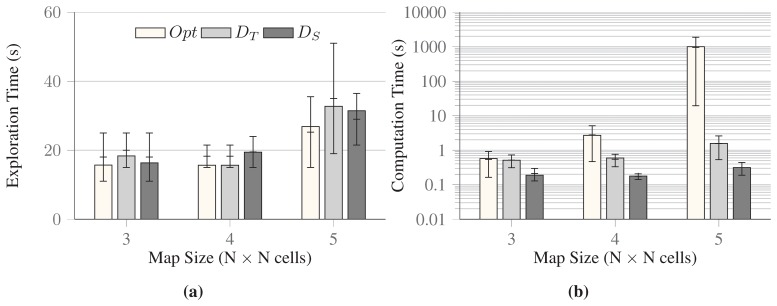
Comparison of all approaches, optimal and disjoint (*Opt*, *D_T_*, *D_S_*), on three sets of maps. Each set contains maps of varying size, from 3 × 3 to 5 × 5. The solid bars indicate the average values over the 3 maps for 3 × 3 and 10 maps for the rest, and error bars show the minimum and maximum values observed during the trial. (**a**) shows the solution quality; and (**b**) shows the computation time taken by the all three approaches on a logarithmic scale.

The evaluation focuses on two aspects: solution quality (in terms of overall exploration time) and time required to compute the solutions. However, finding the optimal solution for maps as small as 6 × 6 becomes already an unpractical task, as the computation of 12 h on an Intel i7 Quad Core 2.60 GHz computer with 8 GB RAM was not enough to find the solution. The aggregated results for the comparison of all three methods up to maps of size 5 × 5 are presented in Figure 6. Here, *Opt* indicates the optimal solution, *D_T_* the decoupled approach in which traveling time is minimized first, and *D_S_* the decoupled approach in which sensing time is minimized first. In particular, Figure 6a illustrates the aggregate quality of the solutions provided by the two decoupled approaches and the optimal one. In this limited set of examples, the disjoint approach where traveling time is minimized first shows better solution quality whenever it is possible to discard high number of redundant sensing configurations along the traveling route, whose field of view overlap. On the other hand, the disjoint approach where sensing time is minimized first shows better solution quality whenever the traveling path along the sensing configurations in the solution is small. When we consider the time necessary to compute a solution, the disjoint approach where the sensing times are minimized first is the most efficient, while the optimal solution becomes soon too expensive to be of any practical use (Figure 6b).

**Figure 7.**
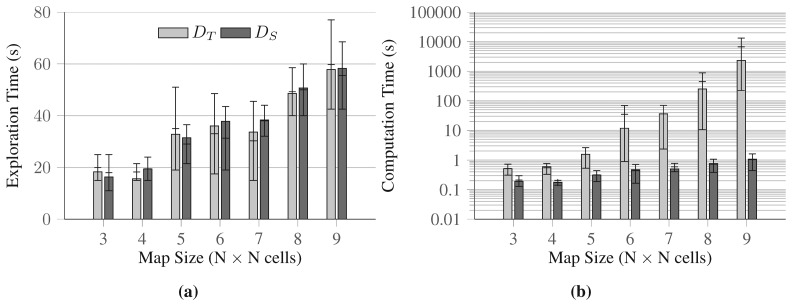
Comparison of two disjoint approaches up to maps of size 9 × 9. Notations are similar as in the previous figure, *i.e.*, bars indicate the average values over the 3 maps for 3 × 3 and 10 maps for the rest, and error bars indicate the minimum and maximum values observed during the trial. (**a**) shows the solution quality of two disjoint approaches (*D_T_*, *D_S_*); and (**b**) shows the average computation time on a logarithmic scale for both approaches.

We tested the decoupled approaches on the remaining 4 sets of maps (of size 6 × 6 to 9 × 9), and the results are presented in Figure 7. Since we could not calculate the optimal solutions for those runs, we directly compare the quality of the solutions found by the two disjoint approaches. In this second set of tests, *D_T_*, in average, generates solutions of slightly better quality (Figure 7a), that is, solutions where the overall exploration times are lower. On the other hand, the computation times for this decoupled approach grow quickly as the size of the maps increase, as it is clearly shown in Figure 7b. It is very important to note that 9 × 9 maps are still not large enough for representing real-world problems, and that *D_T_* requires a time in the order of hours to solve such simple instances. On the other hand, *D_S_* generates solutions for the same instances in less than two seconds, with a reasonable loss of quality (in this set of experimental runs, between 0.69% and 13.81% to the *D_T_*).

We continued our tests focusing on *D_S_* up to maps of size 26 × 26 when also this decoupled approach begins to require computation times in the order of hours. The bottleneck of the computation lies in finding a set of minimum cost candidate sensing configurations which can cover the whole map. This is a Sensor Placement Problem (SPP), which has been studied extensively in the past decades [27]. In the following, we propose a new convex relaxation method which allows us to drastically curtail the computation times.

## 7. A New Re-Weighted Convex Relaxation for Solving Sensor Placement Problems

To solve the problem of finding the set of sensing configurations that covers the whole environment at minimum cost (as we do in the decoupled approach described in Section 5.2) for instances with a high number of variables, we propose an iterative convex relaxation method which introduces intense sparsity, thus drastically reducing the number of variables to consider. The reduced problem can then be cast into an integer linear programming problem as formulated in Equation (5).

**Figure 8.**
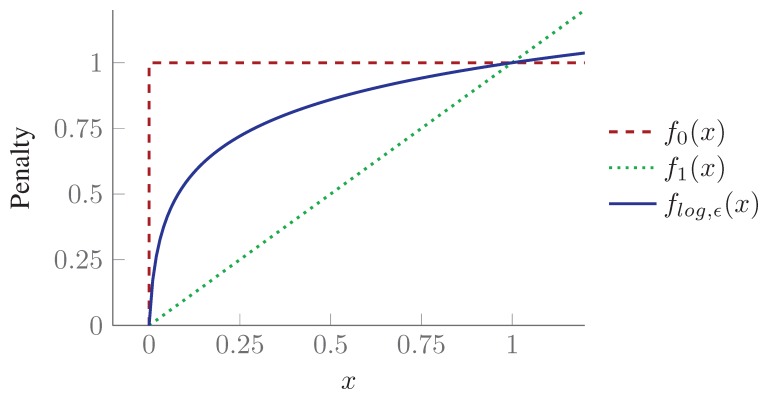
The concave loss function *f_log,ϵ_*(*x*) approximates better the ℓ_0_ sparsity count *f*_0_(*x*) than by the traditional convex ℓ_1_ relaxation *f*_1_ (*x*) [48].

We call this algorithm *SPP with Re-weighted Convex Relaxation*, or in short *conv*-SPP. The SPP described in Equation (5) is NP-hard and corresponds to ℓ_0_-minimization whose solutions are binary variables, *i.e.*, sensing configurations. The combinatorial search soon becomes impracticable as the size of the problem grows. It is because ℓ_0_-minimization penalizes non-zero elements identical and the overall solution cost is based on the cardinality of the set. ℓ_1_-minimization on the other hand is much faster than ℓ_0_-minimization, and therefore, can handle relatively high number of variables. However, it penalizes each element proportional to the magnitude and thus the solution is no longer a binary vector (see Figure 8).

Candès *et al.* [48] proposed a re-weighted (iterative) formulation of ℓ_1_-minimization to handle large instances of a discrete problem in continuous domains. The approach effectively uses a concave loss function (*f_log,ϵ_*(*x*)), which is much closer to the penalty function of ℓ_0_-minimization (see *f*_0_(*x*) in Figure 8). The essential part of the approach is to solve an overall non-convex optimization problem through iterations of convex optimization. To sparsify the solution vector along the curve *f_log,ϵ_*(*x*) in Figure 8, a weight vector is introduced which evolves iteratively. The resultant vector is much sparsifer than the result of simple ℓ_1_-minimization with a linear penalty function. Afterwards, the reduced set of non-zero elements forms a reduced search space in which the combinatorial problem in its integer linear formulation (Equation (5)) can be solved in a feasible time.

Formally, our *conv*-SPP formulation, inspired by [48] is:

(6a) $$\begin{array}{l} \underset{C}{\mathrm{minimize}}{\left(W\circ C\right)}^{T}T_{s}\\ \text{subject to} \end{array}$$

(6b) VC≽1

(6c) 0≼C≼1

*W* is a weight vector of cardinality | 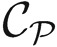
|. At the beginning, all the elements of *W* are equal to 1, *i.e.*, *W* = 1. In subsequent iterations, the weights are updated according to Equation (7).

(7) $$\begin{array}{cc} W_{\left(i\right)}=\frac{\in }{C_{\left(i\right)}+\in } & i=1,\ldots ,\left|W\right| \end{array}$$

**Figure 9.**
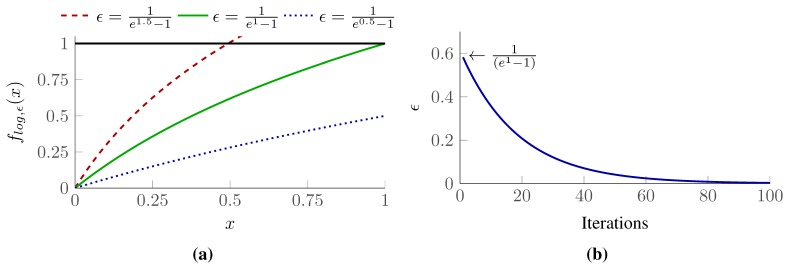
(**a**) Smaller value of *ϵ* defines steeper penalty functions; (**b**) The exponential decay of *ϵ* during the iterative procedure.

The parameter *ϵ* is strictly positive and determines the rate of convergence. Smaller values of *ϵ* allow for faster convergence (see Figure 9a) but at an increased risk of getting trapped in a local-minimum.

**Figure 10.**
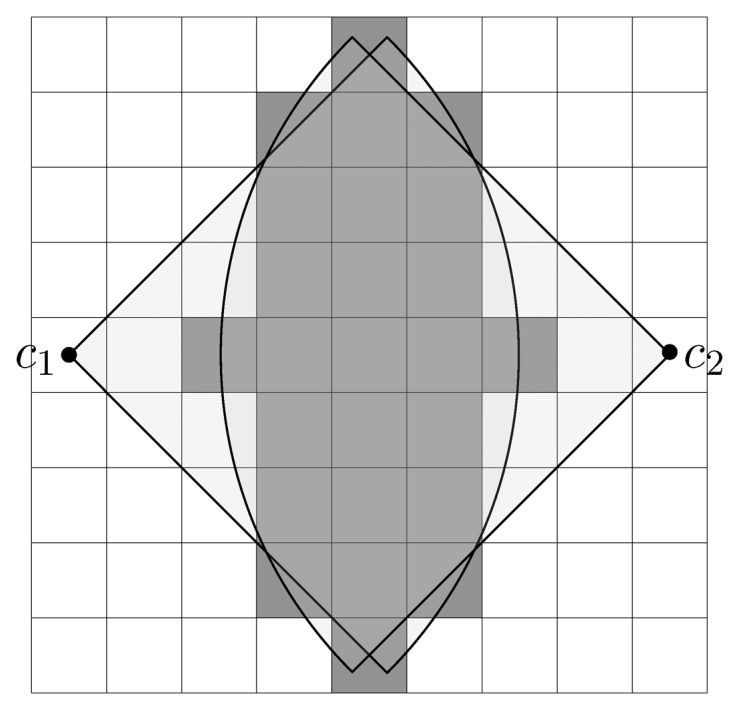
The sensing configurations *c*_1_ and *c*_2_ are trapped in a local minimum. The cells under the overlapping field of view are indicated in gray. Assuming that the remaining visible cells of both configurations, indicated in white under the coverage window, are partially (50%) observed by another configuration(s) in the solution. The *c*_1_ and *c*_2_ are set to 0.5 in the solution vector and any subsequent iteration does not improve the situation.

A local-minimum may occur whenever two or more variables have identical values and none of them is converging to the maximum or minimum limit. This could happen, for example, when two or more candidate sensing configurations have overlapping field of view and share a common subset of coverage. In Figure 10, *c*_1_ and *c*_2_ are two sensing configurations facing each other. The set 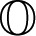 contains elements (cells) under the overlapping field of view, i.e. 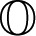 ⊂ *υ*_
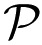
_(*c*_1_) and 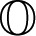 ⊂ *υ*_
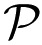
_(*c*_2_). If *υ*_
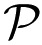
_(*c*_1_) \ 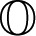 and *υ*_
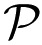
_(*c*_2_) \ 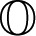 are partially observed by another selected sensing configuration(s) in the solution. The configurations *c*_1_ and *c*_2_ will hold half of the magnitude to minimize the overall solution cost in continuous domain, and any further iteration will not improve the situation, since the algorithm can not decide which of the two sensing configurations it prefers. This problem has then to be resolved in the subsequent combinatorial optimization step.

In our implementation we adapt the value of *ϵ* in each step *i* according to Equation (8), so that it sets relatively high value at beginning to avoid a local minimum in early iterations to explore globally better solution, and then deceases to exponential decay with each iteration to speed up the convergence (see Figure 9b).

(8) $$\in_{\left(i\right)}={\left(\frac{1}{e^{1}-1}\right)}^{1+\left(\left(i-1\right)\times 10^{-1}\right)}$$

The procedure stops if no improvement in sparsity for the last *n* (*n* = 5 in our implementation) iterations is observed, or if a predefined number of iterations (150 in our case) is reached. We use $l_{\tau }^{0}$ sparsity measure with *τ* equals 10^−2^. In addition, we stop if the number of non-zero elements is low enough so that the combinatorial search is feasible (the value was empirically set to 80). Finally, the reduced vector of candidate sensing configurations in *C* is solved with the combinatorial method according to Equation (5). The overall procedure is summarized in Algorithm 1.



**Algorithm 1**
*conv*-SPP
1:Set *W* = 1;2:Solve Equation (6);3:Update *W* as in Equation (7) and *ϵ* as in Equation (8);4:Go back to step 2, if none of the stopping criteria is true;5:Discard zero elements of *C*;6:Solve Equation (5) with updated *C*;


In addition to the solution itself, our algorithm provides an interval in which the optimal solution for the sensing time minimization lies. The upper bound for this interval is the found solution of Algorithm 1, since the optimal solution is either equal to our solution or faster. The lower bound is obtained by considering the continuous problem instead of the combinatorial one. Simple ℓ_1_-minimization with linear penalty function *f*_1_(*x*); which is the first iteration of step 2 in Algorithm 1, will provide a solution, which is always less than or equal to the optimal solution of combinatorial search in terms of sensing time.

The limits can be used to assess the quality of the solution when the optimal solution is not available. However, in practice, the optimal sensing time is very close to the upper bound of *conv*-SPP, as shown in the following section (Section 8).

## 8. Evaluation of the New Method

We evaluated *conv*-SPP on sets of randomly generated grid maps of varying size, from 3 × 3 to 90 × 90 cells. In each map, 10% of the cells are occupied by obstacles, and we assume that cells have an identical side length of 1 m. Each set is composed by 10 different maps of identical size, with the only exception of the set of maps of size 3×3, where, considering all symmetries, only 3 unique instances are possible.

In each test run, the candidate sensing configurations are defined such that $\Theta =\left\{0,\frac{\pi }{2},\pi ,\frac{3}{2}\pi \right\}$, and *ϕ* and *r* are fixed. Therefore, the sensing time for each candidate sensing configuration is set to 1 and the algorithm minimizes the number of sensing configurations. To evaluate the dependency of the algorithm with respect to the sensing parameters, we consider two different sensing radii (*r* = {15, 30} meters) and two central angles ( $\phi =\left\{\frac{\pi }{2},\pi \right\}$).

To solve the optimization problem, we used the Gurobi Solver [49], with CVX, a package for specifying and solving convex programs [50,51] in Matlab. For these runs, we used a computer with an Intel i7 Quad Core 2.60 GHz and 8 GB RAM.

We compared the optimal solutions of the sensor placement problem instances with the solutions provided by *conv*-SPP on maps up to the size of 26 × 26 cells. Larger maps required computation times in the order of hours to solve a single instance optimally.

**Figure 11.**
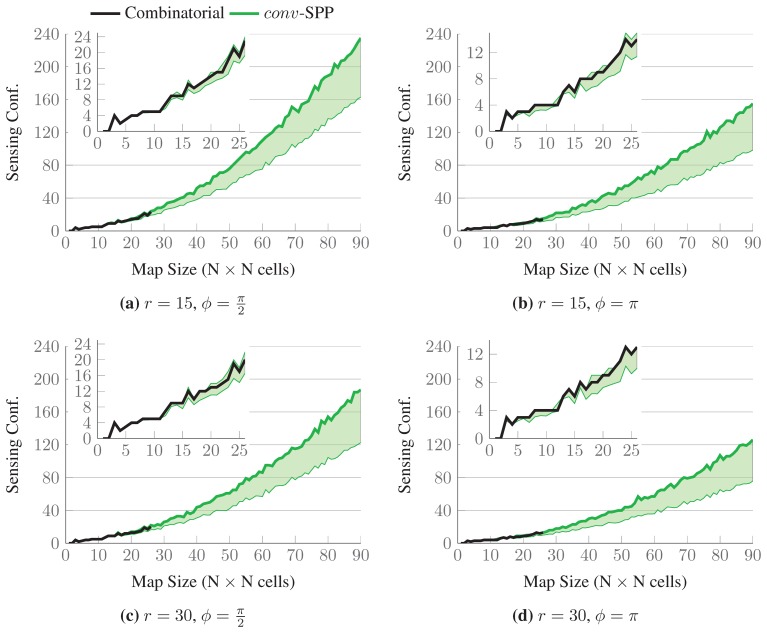
Comparison of the quality of the solutions obtained with *conv*-SPP and the combinatorial method on randomly generated maps. Sensing parameters are fixed to $\Theta =\left\{0,\frac{\pi }{2},\pi ,\frac{3}{2}\pi \right\}$, *r* = {15, 30}, and *ϕ* = {*π*,*π*/2}. The optimal solutions (thick black lines) are close to the solutions provided by *conv-SPP* (thick green lines) with respect to the number of sensing configurations selected. The lower bounds of the green intervals represent the result of a single ℓ_1_-minimization step.

**Figure 12.**
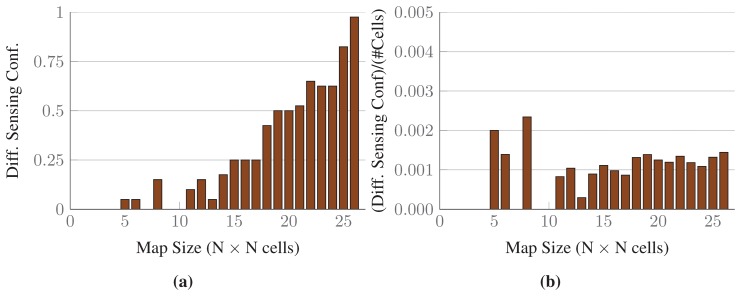
The solution quality gap between *conv-SPP* and combinatorial optimization. (**a**) shows the average difference for all possible combinations of the sensing parameters *r* = {15, 30} and *ϕ* = *{π*, *π*/2}; (**b**) shows the average difference divided by the number of cells in the maps.

The results for a single map randomly chosen from each set are shown in Figure 11. The thick black lines represent the number of sensing configurations selected in the optimal solutions, while the upper bounds of the green-colored intervals represent the number of sensing configurations selected by *conv*-SPP. The lower bounds of the intervals represent the number of configurations after the first iteration of the ℓ_1_-minimization. The inset graphs show the results up to maps of size 26 × 26, for which the optimal solutions are available. *conv*-SPP obtains results which are very close to the optimal solutions in terms of the number of sensing configurations selected, as our method does not add more than two sensing configurations for any instance. Figure 12 shows the average difference of solution quality, which, for the available results, is always less than one sensing configuration.

**Figure 13.**
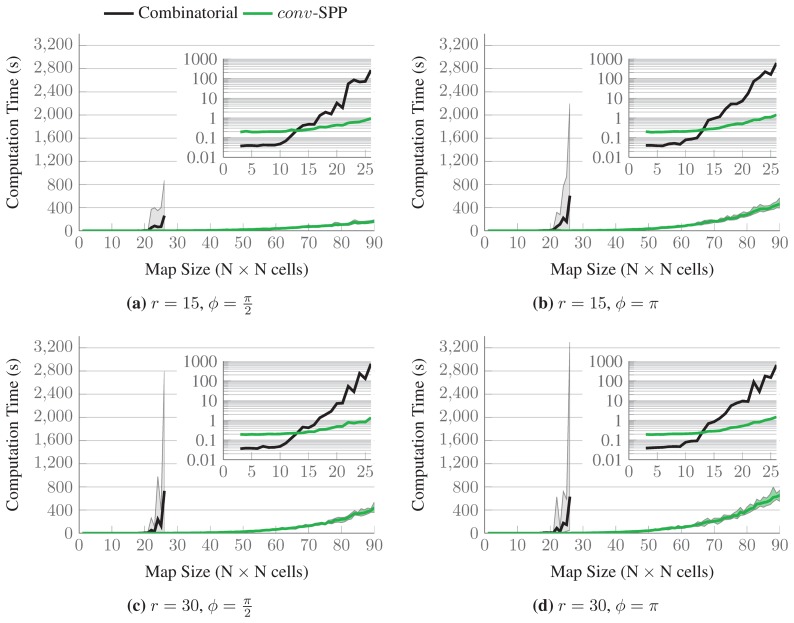
Computation times to calculate the optimal solutions and to solve the instances with *conv*-SPP. The thick lines represent the average computation times for each set of maps, while the colored intervals are bounded by the minimum and maximum times it took to solve all the instances in one set. The inset graphs show the computation times on a logarithmic scale.

Figure 13 shows the aggregated computation times for both approaches: The thick lines represent the average computation times for each set of maps, while the colored intervals are bounded by the minimum and maximum times it took to solve all the instances in one set. The inset graphs show the computation times on a logarithmic scale: As it can be seen, the time required to calculate the optimal solution of the problem increases steeply with the size of the maps considered. It is worth noticing that the calculation of the optimal solutions for maps up to 13 × 13 cells takes less time than when *conv*-SPP is employed. This is because the number of variables is small and the combinatorial method can find a solution quickly. On the other hand, *conv*-SPP runs at least one iteration of convex relaxation and then performs combinatorial optimization for the remaining variables. Therefore, the iterations of convex relaxation simply require additional time. We stopped computing the optimal solutions for a set of maps whenever at least one instance of the set required more than one hour to be solved optimally. By contrast, our method could solve all the instances in less than 800 s.

Within the same set of maps of identical size, different instances can require very different computation times for computing the optimal solution. Conversely, this is not the case when using *conv*-SPP. This is because few changes in the map layout can completely change the discrete optimization problem, while, on the other hand, the continuous problem is much less affected, and in *conv*-SPP the combinatorial solver is used only on a reduced set of variables. Hence, another advantage of *conv*-SPP is that the computation time is largely independent of the particular problem instance within a given problem size.

**Figure 14.**
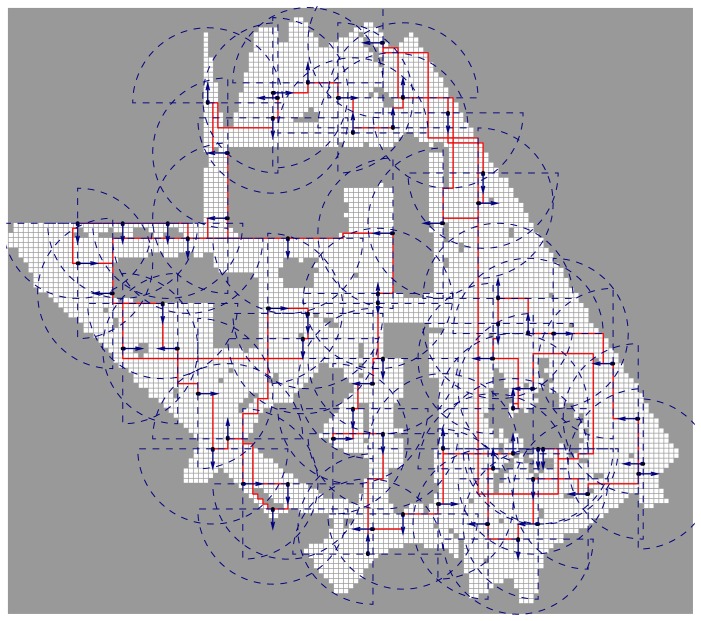
The exploration plan generated for the map of the Freiburg University campus (obstacles are represented in gray). The positions of the sensing configurations are represented with blue dots, blue arrows indicate their orientation and dashed-lines their field of view. A closed path through the configurations is shown in red.

**Figure 15.**
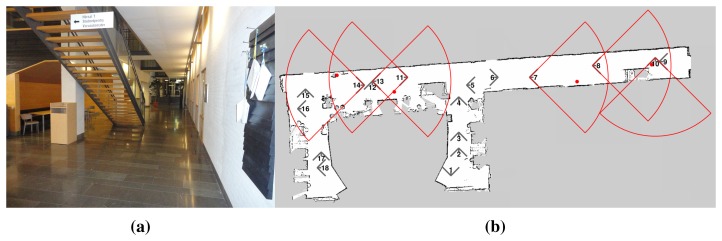
(**a**) an indoor setting for the gas detection task; (**b**) graphical results: the gas sources are indicated by red dots, the planned sensing configuration by truncated cones in gray, and the small number associated to each configuration indicates the order in which they are visited. The configurations plotted in red indicate the positive readings and the actual area they cover.

We also used the Freiburg University campus map [54] to demonstrate our algorithm on a real map. We discretized the map into a Cartesian grid where each cell covers 1 m^2^. The map size is 120 × 136 cells, 6113 of which are unoccupied (see Figure 14). For the sensing parameters $\Theta =\left\{0,\frac{\pi }{2},\pi ,\frac{3}{2}\pi \right\}$, *r* = 15 m, and *ϕ* = *π*, our algorithm generated a solution of 68 sensing configurations in 90.87 s with a lower bound of 53.03 configurations. This scenario is evaluated in based on real map data (but without actual gas sources) to verify that our approach is able to efficiently cover a large real environment. In our ongoing work, we are performing actual gas detection tasks using the described algorithm on a robot in different environments (see Figure 15a for an example). Initial results are quite promising, as shown in Figure 15b. For more details on the experiments and the evaluation, we refer the reader to our forthcoming publication [52].

## 9. Conclusions

In this paper, we discussed the problem of gas detection in large environments with a mobile robot equipped with a remote gas sensor. Generally speaking, given an environment where a specific gas may be present in detectable concentrations, a solution to the problem would be a cost-effective exploration plan for the robot so that it could ascertain whether this is the case or not. Here, we consider cost to be directly proportional to the time required to complete the exploration plan.

We provided a formal definition of the problem and suggested several possible methods for solving it. The main contribution of this paper is *conv*-SPP, an algorithm for quickly finding an exploration plan which guarantees a complete coverage of the environment. Our algorithm leverages a novel method based on convex relaxation that drastically reduces the number of variables and it is therefore effective for solving large problem instances. Furthermore, *conv*-SPP offers guarantees on the quality of the solution, as it is always possible to asses the theoretical maximum distance of the solution provided to the optimal one. In practice, we show that in all the cases for which we could experimentally solve the problems optimally, our algorithm always generated results very close to the optimal ones.

We performed extensive validation of our approach in simulation, both on randomly generated maps of various sizes and on the map of the Freiburg University campus. We also compared our methods with other approaches, both in terms of solution quality and of computational requirements. In our future work, we want to test our algorithm in real world scenarios with a mobile platform. We also intend to extend our problem definition to take further advantage of remote sensing and include non-traversable but observable cells.

In this article, we are addressing the planning problem for gas detection only. For addressing this problem a pregenerated plan, like the approach we presented here, is sufficient. Future work will address how to deal with actual gas detections. Possible questions of interest include the problem of gas source localization [17,53], which often requires an active strategy to follow the trail to the source. Robot assisted gas tomography for gas distribution mapping with remote sensors [19,46] requires the consideration of different sensing positions in order to meet constraints regarding the overlap of the sensing fields and intersection angles that are necessary to reconstruct the gas distribution. Optimizing the sensing geometry for tomographic measurements [55] will be one focus of our future work. Extending the presented work to multi-robot scenarios, in which a fleet of robots cooperatively monitors a target area [56–60] is another interesting direction for future work.
